# Microstructure and Phase Equilibria in BCC-B2 Nb-Ti-Ru Refractory Superalloys

**DOI:** 10.3390/ma17225429

**Published:** 2024-11-07

**Authors:** Melanie K. Moczadlo, Eric A. Lass

**Affiliations:** Department of Materials Science and Engineering, University of Tennessee Knoxville, Knoxville, TN 37996, USA; mmoczadl@utk.edu

**Keywords:** compositionally complex alloys, high-entropy alloys, refractory superalloys, phase diagrams

## Abstract

Refractory superalloys (RSAs) are promising candidates for high-temperature, high-strength applications. Two-phase RSAs containing body-centered cubic (BCC) and ordered B2 phases are among the more promising candidates. Systems containing Ru-based B2 precipitates exhibit stable two-phase microstructures at temperatures in excess of 1600 °C. The present study experimentally investigated one potential foundational ternary system for these alloys, Nb-Ti-Ru. Two alloys, (Nb_3_Ti)_0.85_Ru_0.15_ and (Nb_4_Ti)_0.85_Ru_0.15_, were studied to determine phase equilibria and properties at temperatures between 900 °C and 1300 °C. The B2 phase was found to be dominated by RuTi ordering, although considerable Nb solubility was observed up to 18 mol %. The Nb-rich BCC matrix contained up to 15 mol % Ru and 20 mol % Ti. Although a two-phase microstructure of B2 precipitates in a BCC matrix was confirmed, the distribution of elements in the two phases resulted in a larger lattice misfit than expected. The results obtained in this investigation provide valuable information for the future development of RSAs utilizing Ru-based B2 strengthening precipitates.

## 1. Introduction

The need for materials to withstand ever more demanding conditions such as in aerospace and nuclear applications has spurred research into new high-temperature classes of alloys. One such emerging class of materials is compositionally complex alloys (CCAs). CCAs are composed of three or more principal elements. The large composition space of CCAs allows considerable design freedom and tailoring of the microstructure and properties [1]. A refractory CCA (R-CCA) is a CCA containing one or more refractory metals. The high melting temperature of refractory elements combined with the unique mechanical properties of CCAs offers great potential to meet the higher temperature demands required for emerging structural applications. Although Mo, Nb, Ta, W, and Re are the primary refractory elements, other Group IV, V, and VI elements (Ti, Zr, Hf, V, and Cr) are often considered to be extended refractory elements [2,3].

Refractory superalloys (RSAs) are a class of materials that are related to R-CCAs, so-called due to their parallels with Ni-based superalloys. RSAs often have the compositional complexity of R-CCAs combined with secondary-phase strengthening precipitates. RSAs consisting of two phases, body-centered cubic (BCC) and ordered B2, have recently gained great interest due to the similarity of their microstructure to γ′-strengthened Ni-based superalloys, the current material of choice for high-temperature applications [3,4,5]. These BCC-B2 RSAs mimic the γ-γ′ microstructure of Ni-based superalloys, consisting of a high-volume fraction of coherent and often cuboidal precipitates in a matrix of a second phase [3,4,5,6,7,8,9,10,11,12,13,14]. Although a microstructure consisting of strong, ordered B2 precipitates in a more ductile BCC matrix (B2-in-BCC) is most desirable, akin to γ′-L1_2_ in γ-FCC in Ni-based alloys, RSAs containing BCC and B2 have more commonly occurred as a co-continuous mixture of the two phases resulting from spinodal decomposition [4,10,11,12,15,16], or as a BCC precipitate in a B2 matrix, or as an “inverted” (BCC-in-B2) microstructure [3,4,9,10,17,18]. Lass recently detailed the thermodynamics of the higher-order BCC-B2 phase relationship in multicomponent alloys, specifically R-CCAs, and demonstrated that although a conventional B2-in-BCC microstructure is possible, the thermodynamics and resulting phase diagrams for multicomponent alloys need to be understood in more detail in order for such regions to be accessible [4].

Although BCC-B2 alloys need to be understood further, there is already some groundwork on experimental BCC-B2 refractory alloys. Senkov et al. began work on multiple Al-containing alloys, including AlMo_0.5_NbTa_0.5_TiZr. This alloy exhibited a BCC-B2 microstructure formed via spinodal decomposition that demonstrated high strength up to 1200 °C but brittle behavior at temperatures below 600 °C [7]. From this alloy, four compositional variations were created in an attempt to reduce brittle behavior at low temperatures, but a good compromise between strength at high temperatures and ductility at low temperatures was not obtained [8]. Soni et al. built on this work and created the BCC-B2 alloy Al_10_Nb_15_Ta_5_Ti_30_Zr_40_, which exhibited better ductility at room temperature, but the formation of Al_x_Zr_y_ intermetallics occurred within the BCC-B2 microstructure [11]. Laube and co-workers identified a potential B2-in-BCC two-phase field in a TaMoCrTiAl system [19,20,21,22] but, like most reported Al-containing BCC-B2 RSAs, the B2 solvus temperature was low, only about 1000 °C, which is lower than many Ni-based superalloys, limiting their potential as next-generation high-temperature materials.

Frey et al. reported a Ru-containing B2 phase in as-cast, equiatomic HfNbTaRuZr that was stable up to >1575 °C [1]. Lass recently suggested that Ru-containing alloys with Group IV and V elements may offer stable BCC+B2 microstructures up to 1600 °C or greater [4,23] and also elucidated the complex thermodynamics of multicomponent BCC-B2 alloy systems, identifying the conditions required to form the desired B2-in-BCC morphology. Kube et al. followed this work by employing high-throughput calculation of phase diagrams (CALPHADs) using ThermoCalc 2023a software to identify potential pseudo-binary alloys in ternary and quaternary systems containing a RuX (where X is Ti, Zr, Hf, or Al) B2 phase and a BCC matrix comprising one or two group V or VI elements (V, Nb, Ta, Cr, Mo, or W) [24]. They experimentally investigated 12 quaternary compositions of Ru_15×15_Z^1^_35_Z^2^_35_ (where X is Ti, Hf, or Al and Z^1^ and Z^2^ are Mo, Nb, Ta, or V) and 6 ternary compositions of Ru_15_X_15_Y_70_ (where X is Ti, Hf, or Al and Y is Nb or Mo). They reported phase compositions, approximate B2 solvus and solidus temperatures, and BCC/B2 lattice misfits, δ, (annealing at 1300 °C), as follows:

(1) $$\delta =\frac{2\left(a_{B2}-a_{BCC}\right)}{\left(a_{B2}+a_{BCC}\right)}$$

where $a_{BCC}$ and $a_{B2}$ are the lattice parameters of BCC and B2, respectively. The ternary Mo and Nb alloys with Ru and Ti had misfits of −2.5% and −5.8%, respectively, while most quaternary alloys exhibited a δ of ±4.5%. Three quaternary alloys, Ru_15_Ti_15_Mo_5_V_35_, Ru_15_Hf_15_Mo_35_Nb_35_, and Ru_15_Hf_15_Mo_35_Ta_35_, were found to have negligible lattice misfits. They concluded that the three systems with low lattice misfits may be promising candidate systems for future BCC-B2-based RSAs.

The systems identified by Kube et al. all contained Mo, which promotes the formation of potentially deleterious phases, including Laves phases with Hf and Zr and a σ-phase with Ru [24]. However, none of the ternary Ru-containing refractory systems have been experimentally studied in detail [25]. The purpose of the present study was to provide a focused investigation into the microstructure and phase equilibria of the ternary Nb-Ti-Ru system to provide the essential experimental data required for the development of thermodynamic databases for the future design of Ru-containing RSAs. This system avoids the potential deleterious phases promoted by Mo, while the relatively low negative lattice misfit between BCC and B2 in Ti-Ru combined with the positive lattice misfit between BCC and B2 in binary Nb-Ru offer potential as a foundation for the development of B2-in-BCC RSAs with a low lattice misfit. The available information on the Nb-Ru binary system reports a higher-order BCC-B2 transition similar to that found in Fe-Al or Fe-Si, with no two-phase BCC+B2 region. A transformation from B2 to an orthorhombic NbRu phase has previously been reported [25] but not yet universally accepted as an equilibrium phase; this is the only phase reported in any of the constituent binary systems other than BCC and B2. The Ti-Ru binary system contains a two-phase BCC-B2 region but no higher-order transition. The reported lattice parameters of BCC Ti-20Ru and RuTi B2 (Ti-50Ru) are 3.161 Å and 3.06 Å, respectively [26], suggesting a δ of −3.25%. On the other hand, the lattice parameters of BCC Nb-35Ru (mol %) and B2 Nb_1.24_Ru_0.76_ (Nb-38Ru) are 3.188 Å and 3.21 Å, respectively [27], for a δ of +0.68%. The Nb-Ru system is the only binary Ru refractory system with a positive lattice misfit. The positive δ of Nb-Ru opens the possibility of decreasing the magnitude of δ in Ti-Ru. Kube et al. reported the limited solubility of Nb in the RuTi-type B2 they found in this ternary system, but if the alloy composition could be modified to alter the partitioning between BCC and B2, a two-phase BCC+B2 microstructure with a low lattice misfit might be possible. Even if a low lattice misfit is not possible in this ternary system, the high B2 phase stability at >1800 °C [4,24] combined with (1) a single-phase BCC phase field above the BCC+B2 region where solutionization can be performed and (2) no (or possibly one) secondary phase that could compete with B2 formation make Nb-Ti-Ru an excellent foundational system for BCC-B2 superalloys and a system that warrants more detailed experimental investigation.

## 2. Materials and Methods

### 2.1. Experimental Design

**Figure 1:**
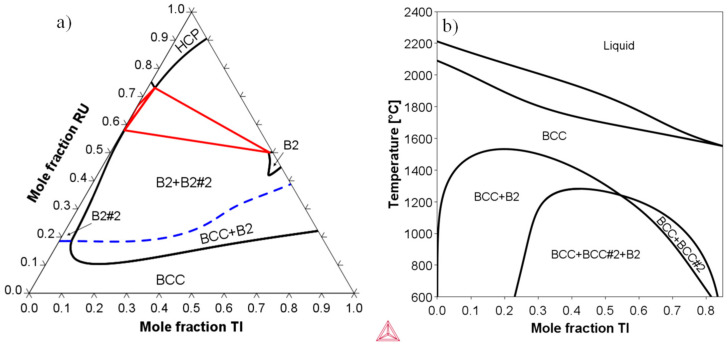
(**a**) Isothermal section at 1400 °C and (**b**) 15 mol % Ru isopleth of the Nb-Ti-Ru system. The dashed blue line in (**a**) represent the trace of *T_c_* for B2#2.

Figure 1a presents the 1400 °C isothermal section of the Nb-Ti-Ru system, calculated using ThermoCalc software and the TCHEA4 thermodynamic database [28,29]. The dashed line is the ordering composition (trace of the critical ordering temperature, *T_c_*, through a ternary isothermal section) for the B2#2 (NbRu-B2) phase. A narrow BCC+B2 two-phase region existed between 10 mol % and 20 mol % Ru for Ti compositions up to about 50 mol %. The Ru content of the alloys studied in this work was fixed at 15 mol % to coincide with this two-phase field and to yield a reasonable volume fraction of B2 of 30–40%. Figure 1b shows the 15 mol % Ru isopleth of the Nb-Ti-Ru system. The B2 solvus temperature, *T*_B2_, was found to be highest (~1500 °C) for a Ti composition between 15 mol % and 20 mol %. Figure 1 also suggests that a miscibility gap existed in the BCC phase for Ti compositions above about 25 mol %, and BCC phase separation into Nb- and Ti-rich phases was expected.

**Figure 2:**
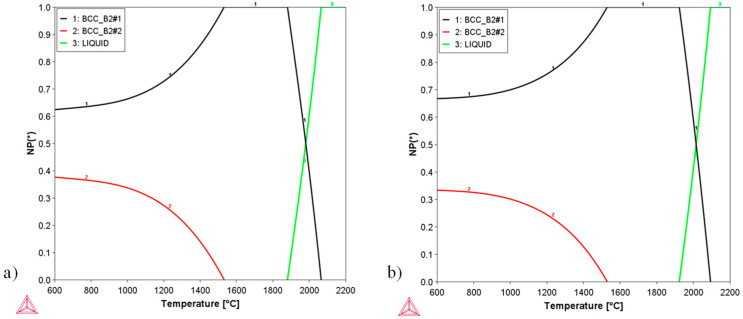
Phase diagrams (phase fraction versus temperature) for the two alloys selected in this work: (**a**) (Nb_3_Ti)_0.85_Ru_0.15_; (**b**) (Nb_4_Ti)_0.85_Ru_0.15_.

As no previous experimental information was available for the ternary Nb-Ti-Ru system, the reported lattice parameters for the two binary Ru-containing systems were used to estimate the lattice misfit between BCC and B2 in the two-phase ternary alloys [26,27]. A rule of mixtures calculation suggested that a Nb:Ti ratio of 4:1 would have a negligible lattice misfit, while a ratio of 3:1 was expected to have a δ=−0.2 %, a typical lattice misfit of Ni-based superalloys. With these considerations, the alloy compositions chosen for this experiment were (Nb_3_Ti)_0.85_Ru_0.15_ and (Nb_4_Ti)_0.85_Ru_0.15_. Figure 2 shows the phase diagrams (phase fraction as a function of temperature) for these two compositions. Both alloys had a *T*_B2_ just above 1500 °C; solidus and liquidus temperatures near 1900 °C and 2100 °C, respectively; and a ~200 °C freezing range. The B2 phase was expected to nucleate from the BCC phase upon formation, evident by a discontinuous composition jump from the parent BCC to the B2 precipitate (Supplementary Figure S1).

### 2.2. Sample Preparation

The two compositions identified above, (Nb_3_Ti)_0.85_Ru_0.15_ and (Nb_4_Ti)_0.85_Ru_0.15_, hereafter referred to as Nb3Ti and Nb4Ti, respectively, were prepared from 99.9+% pure elements by arc melting under an Ar atmosphere, flipping each one a minimum of five times to obtain a homogeneous mixture. At the time the experiments were performed, no furnace was available for homogenizing the alloys above 1500 °C in an inert atmosphere. Therefore, aging experiments were directly performed on as-cast materials at annealing temperatures of 900 °C, 1100 °C, and 1300 °C for times of 1 and 7 days in a tube furnace running under flowing argon. After each heat treatment, the sample was quenched in water. As-cast and annealed samples were mounted and polished to 1 µm using standard metallographic techniques for microstructural examinations and a scanning electron microscope (SEM). Back-scatter electron (BSE) imaging was used for all microscopy because the differences in the Ru content of the different phases provided excellent phase contrasts. Energy dispersive spectroscopy (EDS) was performed to confirm the bulk composition and measure the compositions of individual phases. X-ray diffraction (XRD) was performed using Cu K-α radiation to identify the phases present and determine the lattice parameter of each. Microhardness was ascertained to obtain the aggregate properties of the two-phase microstructure, while nanohardness was used to measure the hardness of the different phases. SEM imaging was then used to determine which indents correlated with which phase.

## 3. Results and Discussion

### 3.1. Microstructure and Compositional Analysis

**Figure 3:**
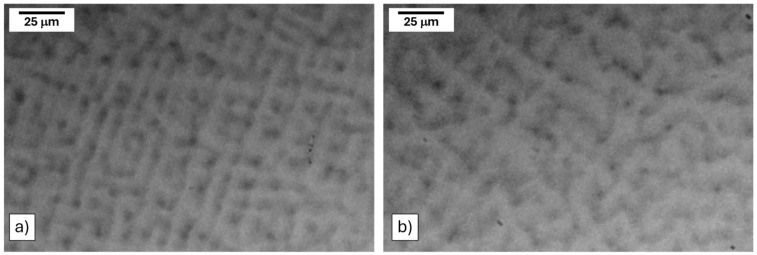
Micrographs of the (**a**) (Nb_3_Ti)_0.85_Ru_0.15_ and (**b**) (Nb_4_Ti)_0.85_Ru_0.15_ alloys in their as-cast condition.

The as-cast microstructures for both alloys are shown in Figure 3. Qualitatively, both alloys were identical, consisting of dendritic solidification with Nb-rich cores (bright) and Ti- and Ru-rich interdendritic regions (dark). This was consistent with the Scheil solidification simulations calculated using ThermoCalc software (Supplementary Figure S2). The Scheil simulation also predicted the formation of a small amount (<5%) of a RuTi-B2 phase, which was not experimentally identified.

**Figure 4:**
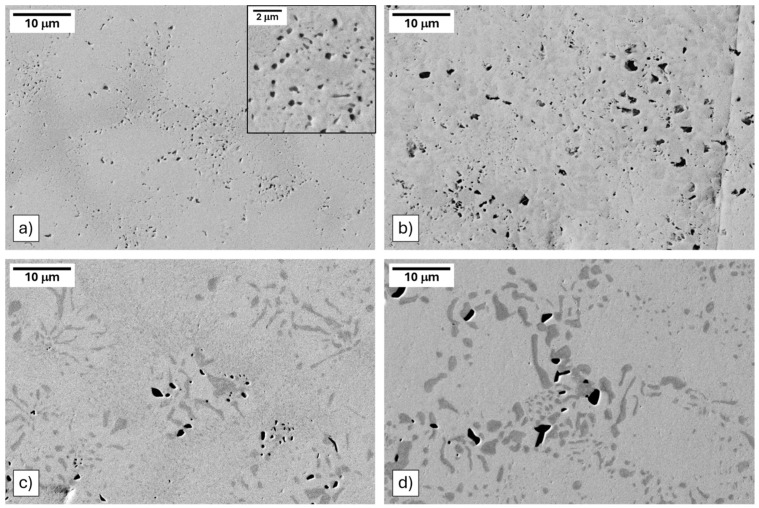
Micrographs of (Nb_3_Ti)_0.85_Ru_0.15_ at different annealing temperatures and times: (**a**) 900 °C for 1 week; (**b**) 1100 °C for 1 week; (**c**) 1300 °C for 1 day; (**d**) 1300 °C for 1 week.

SEM images of Nb3Ti annealed at 1300 °C for one day and at 900 °C, 1100 °C, and 1300 °C for one week are shown in Figure 4. The microstructures of the Nb4Ti alloy appeared to be similar for each annealing and can be found in Supplementary Figure S3. After all annealing conditions, the following three phases were visible: a light-grey matrix, a dark-gray precipitate phase, and a black precipitate phase. Slight changes in the shading of the matrix were observed and were a result of compositional heterogeneity due to solidification segregation.

**Figure 5:**
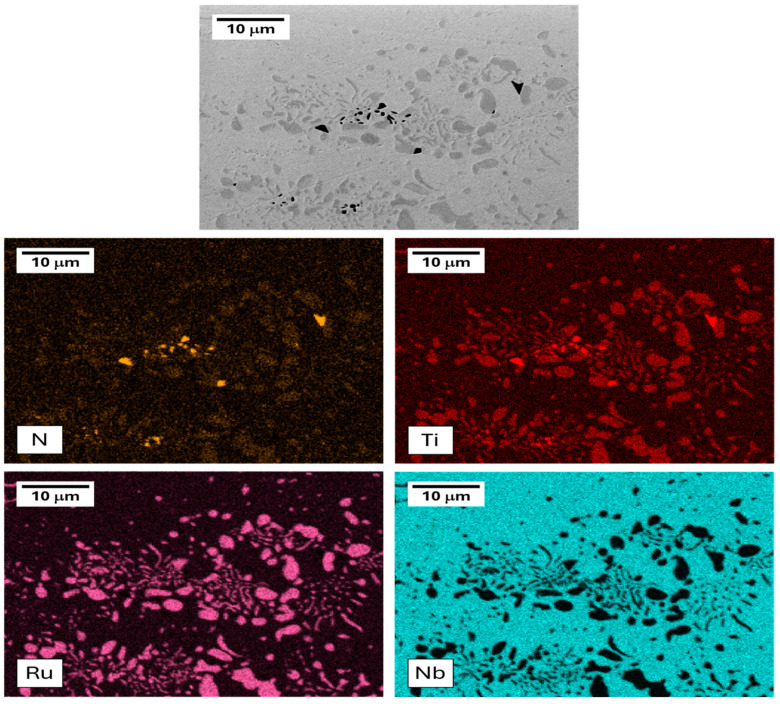
An EDS map of the (Nb_3_Ti)_0.85_Ru_0.15_ sample annealed at 1300 °C for one week showing the N, Ti, Ru, and Nb contents.

As the temperature and/or time increased, both populations of precipitates became larger, making them easier to observe and identify. An EDS map of the Nb3Ti sample annealed at 1300 °C for one week can be seen in Figure 5. The three phases were identified as follows: (1) a light-gray Nb-rich matrix, (2) dark-gray Ti-Ru-rich precipitates, and (3) black titanium nitrides (TiN). The first two phases were expected and were confirmed to be BCC and B2, respectively (below), while the TiN precipitates were likely due to nitrogen contamination of the inert atmosphere during the heat treatment. The presence of TiN precipitates is common in most engineering alloys containing Ti. They are usually highly stable and do not react with the surrounding material. However, Figure 5 shows that the Ti-Ru-rich B2 precipitates were slightly enriched with N, meaning that we could not rule out that the presence of N affected the phase equilibria of Nb-Ti-Ru.

The EDS-measured compositions of the two phases identified in the annealed microstructures are given in Table 1 for both alloys after annealing at 1300 °C for one day with an estimated uncertainty of about ±4.0 mol %. Care was taken to measure only the B2 precipitates that were at least 5 μm in diameter to minimize errors associated with partially measuring the Nb-rich matrix. These precipitates were large enough to obtain reliable composition measurements for the samples annealed at 1300 °C for one day only (the Nb4Ti sample annealed at 1300 °C for seven days was oxidized during annealing). The compositions of the light-gray matrix and dark-gray precipitates suggested they were an Nb-rich BCC matrix and a Ti-Ru-rich B2 phase, respectively, confirmed below via XRD. Although the BCC phase comprised mostly Nb, there was substantial solubility of both Ti and Ru, up to 21 mol % and 15 mol %, respectively. The amount of Ti in the BCC adjusted with the Ti content in the alloy. The amount of Ru in the BCC phase was also found to increase with an increase in the Ti content in the alloy. The B2 phase had a solubility of Nb of 10–20 mol and is slightly off stoichiometry, containing ~43–45 mol % Ru. Despite the formation of both NbRu and RuTi binary B2 phases, the B2 composition in the ternary alloys was found to be dominated by the RuTi B2 phase. However, the observed solubility of Nb in B2 and Ti and Ru in BCC was considerably higher than those reported by Kube at al. [24]. The reason for the high solubility was not clear. It is possible that the N contamination during annealing affected the solubility and equilibrium compositions of the two phases, although the high affinity of Ti for N would suggest that the solubility of Nb in Ti-rich B2 would decrease with the presence of N. A further study of the effects of N (and O) on phase equilibria is needed to elucidate this behavior. Regardless of the reason, these higher-than-expected solubilities opened the possibility of modifying the lattice parameters of the BCC and B2 phases (and the resulting misfits) of two-phase BCC+B2 Nb-Ti-Ru alloys, even before additional alloying elements are introduced.

### 3.2. XRD and Lattice Misfit

**Figure 6:**
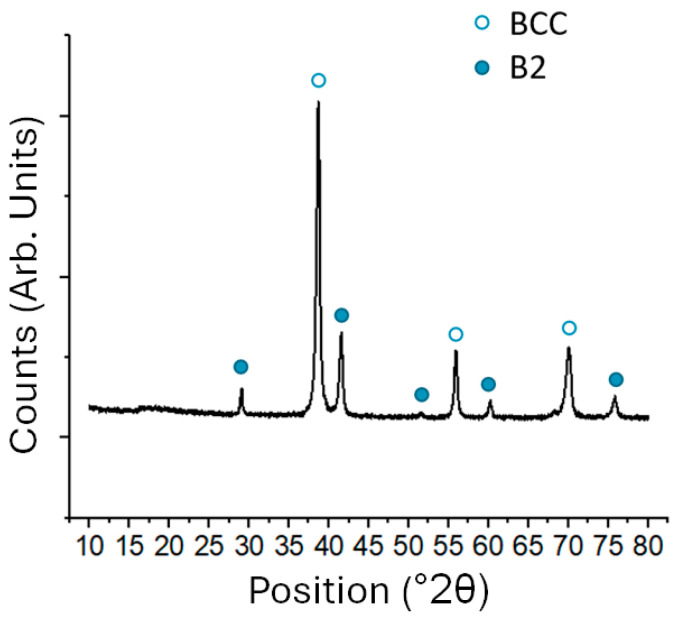
X-ray diffraction results from the (Nb_3_Ti)_0.85_Ru_0.15_ alloy annealed at 900 °C for 7 days.

The XRD results for the Nb3Ti alloy annealed at 900 °C for 7 days are shown in Figure 6. This figure is representative of the XRD data for all annealed samples, with only slight variations in intensity and peak position. There were two sets of peaks indicative of two phases. The peaks at 39°, 56°, and 70° were associated with the BCC phase and the peaks at 29°, 42°, 53°, 60°, and 76° were associated with the B2 phase, confirming a two-phase microstructure of BCC+B2. However, the lattice parameters of the two phases, presented in Table 2, resulted in a lattice misfit much larger than anticipated. The Nb4Ti alloy annealed at 1300 °C for 1 week was destroyed during the annealing process, so no data for that sample were collected. Additionally, the samples annealed at 1100 °C for 7 days were too small to obtain accurate XRD data. All determined lattice misfits were between −5.8% and −6.9%, with the smaller lattice misfits corresponding with the higher temperatures. These misfits were much larger than any previously reported in binary systems between Ru and an extended refractory element, and were consistent with the lattice misfit observed by Kube in Ru_15_Ti_15_Nb_70_ of −5.8% after annealing at 1300 °C [24]. Like the values reported by Kube et al., the BCC and B2 lattice parameters were more in line with those expected for pure BCC Nb and RuTi B2. However, the measured phase compositions showed considerable solubility of all elements in both phases, suggesting that the similarity between the lattice misfits and the binary phases was coincidental, i.e., the increase in the BCC lattice parameter resulting from higher Ti solubility, which is a larger atom than Nb, was offset by the decrease in the lattice parameter resulting from higher Ru solubility, which is smaller than Nb. The molar volumes calculated using ThermoCalc and the TCHEA4 database yielded estimated lattice misfits of −3.0% to −3.5% (see Supplementary Figure S4). The lower lattice misfit calculated by ThermoCalc compared with the experiment appeared to be because of a larger room-temperature lattice parameter (molar volume) for RuTi B2 of 3.17 Å from ThermoCalc compared with 3.06 Å reported in the literature [26]. However, the lattice misfit calculated using ThermoCalc increased with a decrease in temperature, consistent with the experimental results. This considerable change in lattice misfits between the two phases with changing temperatures pointed to an additional issue that needs to be addressed in the future development of BCC-B2 RSAs, namely, interface coherency and stability.

### 3.3. Hardness

Vickers microhardness data for all samples are presented in Table 3. The as-cast samples were clearly the hardest, likely due to a combination of residual stresses from casting/solidification and the solid-solution strengthening of the as-cast BCC single phase. Among the annealed specimens, there was no clear relationship between the microhardness and annealing condition. Samples annealed at 1100 °C were the softest, while those annealed at 900 °C and 1300 °C had approximately the same hardness. The reason for this was unclear, but there were several competing factors that may have affected microhardness as a function of the annealing temperature. First, the compositionally heterogeneous solidification microstructure resulted in a relatively high variability, evidenced by the measurement standard deviations that reached >20 VHN (Vickers hardness number). The 900 °C samples were most likely harder than those annealed at 1100 °C because of the much finer distribution of the B2 phase. The increase in hardness when the annealing temperature was raised to 1300 °C was somewhat surprising considering that it should have had a lower fraction of B2. This may be explained by an increased nitrogen solubility picked up by the BCC matrix at 1300 °C compared with the lower temperatures. There was also no statistically significant difference in the measured microhardness between the two compositions investigated.

Nanoindentation measurements performed on Nb3Ti annealed at 1300 °C for 7 days suggested that the B2 phase was only 4–5% harder than the BCC matrix at 609 ± 36 HV compared with 583 ± 24 HV (note that due to differences between the Vickers microhardness and nanoindentation, there was no one-to-one correlation). The indentations were approximately the same size as the B2 precipitates, so this measured nanoindentation hardness of B2 was likely lower than the actual value and closer to a composite hardness than the single-phase B2. Although flawed because of the heterogeneous composition and precipitate distributions, these hardness measurements suggested that the B2 phase may have provided an increase in strength to the two-phase alloy and the overall strength of the Nb-Ti-Ru two-phase alloys could be comparable to Ni-based superalloys [30,31,32].

### 3.4. Phase Equilibria

**Figure 7:**
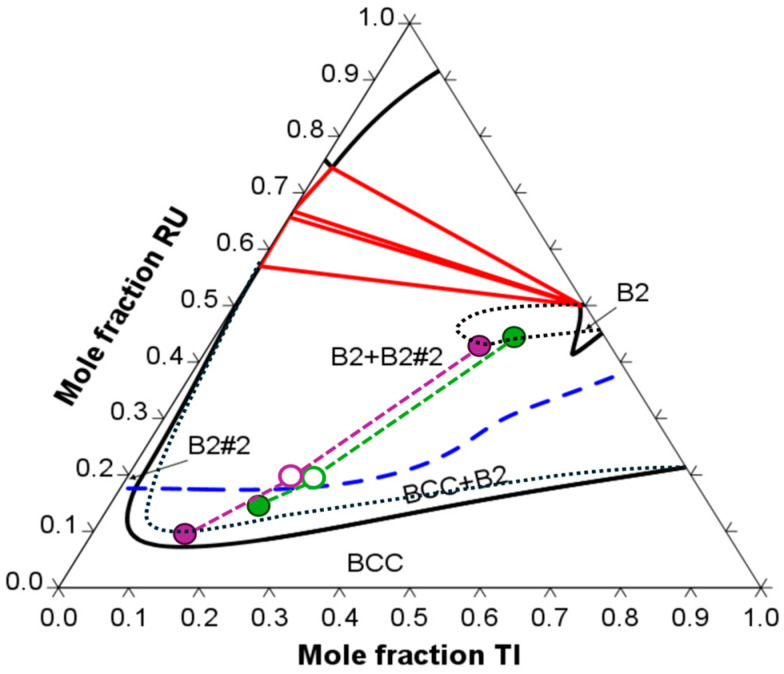
Calculated isothermal section at 1300 °C of the Nb-Ti-Ru phase diagram with the experimentally measured equilibrium tie lines (circles) and updated phase boundaries (dotted black lines). The dashed blue line is the trace of *T_c_* for the B2#2 phase.

Figure 7 presents the 1300 °C isothermal section of the Nb-Ti-Ru phase diagram, calculated using the TCHEA4 database, along with the equilibrium tie lines experimentally measured in the present work. Because of the heterogeneous composition of the characterized materials, the “bulk” composition presented in Figure 7 (open circles) were the measured interdendritic compositions, i.e., the regions of the two-phase BCC+B2 seen in Figure 4 and Figure 5. A minimum of five interdendritic regions were isolated and measured via EDS to obtain average interdendritic compositions, which are presented in Table 1 along with the bulk composition and individual phase compositions (filled circles in Figure 7). Possible adjustments to the calculated phase boundaries are also shown in Figure 7 as dotted black lines. The trace of the critical ordering temperature (dashed blue line in Figure 7) for the NbRu-type B2 phase (B2#2) was not confirmed, although it may be important for potential future applications of alloys based on this or similar systems. Although the boundary between BCC and BCC+B2 shifted with slightly higher Ru contents, the most striking difference between the calculated phase diagram and the measured phase compositions was the solubility limits of the RuTi B2 phase field, which stretched toward the middle of the phase diagram with a constant Ru content of 45–50 mol %, a consequence of the considerable solubility of Nb in RuTi B2 of up to 18.4 mol %. This was in contrast to the Nb content of the B2 phase reported by Kube et al. for Ru_15_Ti_15_Nb_70_ of 5 mol % Nb [24]. However, the expanded B2 phase field shown in Figure 7 was similar to those reported in the related systems for Nb-Ru-Zr [25,33,34] and Hf-Ru-V [35]. The Nb content in the B2 phase did not significantly alter the lattice parameter (3.07 Å in the present work compared with 3.06 Å reported for binary RuTi B2 [26]). However, it did demonstrate that the chemistry of the B2 phase was not fixed and could be tailored, either to manipulate the lattice parameter and misfit or to modify the properties of the B2 phase, such as the stacking fault and/or anti-phase boundary energy, which would ultimately change the mechanical behavior of the B2 phase and the bulk alloy.

## 4. Conclusions

In this experiment, two alloys, (Nb_3_Ti)_0.85_Ru_0.15_ and (Nb_4_Ti)_0.85_Ru_0.15_, were experimentally investigated to determine the phase equilibria and compositions, lattice misfit, and preliminary mechanical properties in the form of micro- and nanohardness. Although a two-phase B2-in-BCC microstructure was confirmed at temperatures between 900 °C and 1300 °C, a large lattice misfit existed between the two phases. However, considerable solubility of Nb was found in the B2 phase of up to 18 mol %, in contrast to the ~5 mol % observed in previous investigations. The solubility of Ru and Ti in BCC was also found to be slightly higher than previously reported, at 15 mol % and 20 mol %, respectively. These high solubilities may prove useful for future alloys in tailoring the misfits or properties of more complex alloys. The identification of TiN in the annealed samples demonstrated the sensitivity of this alloy system to environmental effects, and the role played by N and other interstitials (C and O) in phase equilibria should be studied further. Although the microhardness measurements showed no clear trend with the annealing temperature and time (due, in part, to a heterogeneous microstructure), nanoindentation suggested a potential strengthening effect, even for a non-optimized microstructure and a likely incoherent precipitate–matrix interface. The experimental data collected in this work are valuable for future thermodynamic assessments of the ternary system and the development of an RSA database; however, additional alloying with elements such as Hf, Al, Mo, or V are required to engineer a low misfit precipitate–matrix interface for maximum strengthening and creep resistance.

## Figures and Tables

**Table 1:** Measured compositions after annealing at 1300 °C for 1 day. The bulk and interdendritic compositions are averages from the measurements of all samples.

(Nb_3_Ti)_0.85_Ru_0.15_			
	Ti (mol %)	Nb (mol %)	Ru (mol %)
Bulk	23.5	60.1	16.4
Interdendritic	26.6	54.1	19.3
B2	42.5	12.5	45.0
BCC	21.3	63.9	14.8
(Nb_4_Ti)_0.85_Ru_0.15_			
	Ti (mol %)	Nb (mol %)	Ru (mol %)
Bulk	18.2	67.0	14.8
Interdendritic	23.4	56.8	19.8
B2	38.8	18.4	42.8
BCC	13.3	77.5	9.3

**Table 2:** Lattice parameters *a* (Å) for the BCC and B2 phases and misfits for various annealing times and temperatures.

(Nb_3_Ti)_0.85_Ru_0.15_						
	As Cast	900 °C 1 Day	900 °C 1 Week	1100 °C 1 Day	1300 °C 1 Day	1300 °C 1 Week
B2	-	3.07	3.07	3.07	3.07	3.08
BCC	3.25	3.28	3.28	3.27	3.26	3.27
δ	-	−6.9%	−6.8%	−6.5%	−6.0%	−6.2%
(Nb_4_Ti)_0.85_Ru_0.15_						
	As Cast	900 °C 1 Day	900 °C 1 Week	1100 °C 1 Day	1300 °C 1 Day	1300 °C 1 Week
B2	-	3.07	3.07	3.07	3.08	-
BCC	3.26	3.28	3.28	3.27	3.26	-
δ	-	−6.8%	−6.7%	−6.2%	−5.8%	-

**Table 3:** Measured microhardness for all samples (VHN).

	As Cast	900 °C 1 Day	900 °C 7 Days	1100 °C 1 Day	1100 °C 7 Days	1300 °C 1 Day	1300 °C 7 Days
(Nb_3_Ti)_0.85_Ru_0.15_	477 ± 8	409 ± 17	407 ± 22	358 ± 11	368 ± 15	411 ± 9	395 ± 6
(Nb_4_Ti)_0.85_Ru_0.15_	471 ± 7	424 ± 14	394 ± 25	378 ± 22	372 ± 18	412 ± 11	-

## Data Availability

The original contributions presented in the study are included in the article/Supplementary Materials, further inquiries can be directed to the corresponding author.

## Supplementary Materials

The following supporting information can be downloaded at: https://www.mdpi.com/article/10.3390/ma17225429/s1, Figure S1: ThermoCalc-calculated BCC (solid) and B2 (dashed) compositions for (Nb_3_Ti)_0.85_Ru_0.15_ (left) and (Nb_4_Ti)_0.85_Ru_0.15_ (right) as a function of composition. Black is Nb, green is Ti, and red is Ru. Figure S2: ThermoCalc-calculated Scheil solidification simulations for (Nb_3_Ti)_0.85_Ru_0.15_ (left) and (Nb_4_Ti)_0.85_Ru_0.15_ (right). Top images are temperature versus fraction solidified; bottom images are fraction of each phase versus fraction solidified. BCC_B2#2 is the RuTi-rich B2 phase. Figure S3: Micrographs of (Nb_4_Ti)_0.85_Ru_0.15_ at different annealing temperatures and times: (a) 900 °C for one week; (b) 1100 °C for 1 week; (c) 1300 °C for 1 day. Figure S4: ThermoCalc calculated molar volumes of the BCC (BCC_B2#1) and B2 (BCC_B2#2) phases as a function of temperature for (Nb_3_Ti)_0.85_Ru_0.15_ (left) and (Nb_4_Ti)_0.85_Ru_0.15_ (right).

## References

[1] Frey C., Silverstein R., Pollock T.M. (2022). A high stability B2-containing refractory multi-principal element alloy. Acta Mater..

[2] Miracle D.B., Senkov O.N. (2017). A critical review of high entropy alloys and related concepts. Acta Mater..

[3] Senkov O.N., Miracle D.B., Chaput K.J., Couzinie J.-P. (2019). Development and exploration of refractory high entropy alloys—A review. J. Mater. Res..

[4] Lass E.A. (2022). On the Thermodynamics and Phase Transformation Pathways in BCC-B2 Refractory Compositionally Complex Superalloys. Metall. Mater. Trans. A.

[5] Miracle D.B., Tsai M.-H., Senkov O.N., Soni V., Banerjee R. (2020). Refractory high entropy superalloys (RSAs). Scr. Mater..

[6] Senkov O.N., Woodward C., Miracle D.B. (2014). Microstructure and Properties of Aluminum-Containing Refractory High-Entropy Alloys. J. Miner. Met. Mater. Soc..

[7] Senkov O., Isheim D., Seidman D., Pilchak A. (2016). Development of a Refractory High Entropy Superalloy. Entropy.

[8] Senkov O.N., Jensen J.K., Pilchak A.L., Miracle D.B., Fraser H.L. (2018). Compositional variation effects on the microstructure and properties of a refractory high-entropy superalloy AlMo_0.5_NbTa_0.5_TiZr. Mater. Design.

[9] Soni V., Senkov O.N., Gwalani B., Miracle D.B., Banerjee R. (2018). Microstructural Design for Improving Ductility of an Initially Brittle Refractory High Entropy Alloy. Sci. Rep..

[10] Soni V., Gwalani G., Senkov O.N., Viswanathan B., Alam T., Miracle D.B., Banerjee R. (2018). Phase stability as a function of temperature in a refractory high-entropy alloy. J. Mater. Res..

[11] Soni V., Senkov O.N., Couzinie J.P., Zheng Y., Gwalani B., Banerjee R. (2020). Phase stability and microstructure evolution in a ductile refractory high entropy alloy Al_10_Nb_15_Ta5Ti_30_Zr_40_. Materialia.

[12] Whitfield T.E., Pickering E.J., Christofidou K.A., Jones C.N., Stone H.J., Jones N.G. (2020). Elucidating the microstructural development of refractory metal high entropy superalloys via the Ti-Ta-Zr constituent system. J. Alloys Compd..

[13] Whitfield T.E., Stone H.J., Jones C.N., Jones N.G. (2021). Microstructural Degradation of the AlMo_0.5_NbTa_0.5_TiZr Refractory Metal High-Entropy Superalloy at Elevated Temperatures. Entropy.

[14] Whitfield T.E., Wise G.J., Pickering E.J., Stone H.J., Jones N.G. (2021). An Investigation of the Miscibility Gap Controlling Phase Formation in Refractory Metal High Entropy Superalloys via the Ti-Nb-Zr Constituent System. Metals.

[15] Li J.L., Li Z., Wang Q., Dong C., Liaw P.K. (2020). Phase-field simulation of coherent BCC/B2 microstructures in high entropy alloys. Acta Mater..

[16] Whitfield T.E., Pickering E.J., Owen L.R., Jones C.N., Stone H.J., Jones N.G. (2020). The effect of Al on the formation and stability of a BCC-B2 microstructure in a refractory metal high entropy superalloy system. Materialia.

[17] Allen S.L., Cahn J.W. (1975). Coherent and incoherent equilibria in iron-rich iron-aluminum alloys. Acta Metall..

[18] Soni V., Gwalani B., Alam T., Desari S., Zheng Y., Senkov O.N., Miracle D., Banerjee R. (2020). Phase inversion in a two-phase, BCC+B2, refractory high entropy alloy. Acta Mater..

[19] Laube S., Schellert S., Tirunilai A.S., Schliephake S., Gorr B., Christ H.-J., Kauffmann A., Heilmaier M. (2021). Microstructure tailoring of Al-containing compositionally complex alloys by controlling the sequence of precipitation and ordering. Acta Mater..

[20] Chen H., Kauffman A., Seils S., Boll T., Liebscher C.H., Harding I., Kumar K.S., Szabó D.V., Schlabach S., Kauffmann-Weiss S. (2019). Crystallographic ordering in a series of Al-containing refractory high entropy alloys Ta-Nb-Mo-Cr-Ti-Al. Acta Mater..

[21] Schliephake D., Medvedev A.E., Imran M.K., Obert S., Fabijanic D., Heilmeier M., Molotnikov A., Wu X. (2019). Precipitation behavior and mechanical properties of a novel Al_0.5_MoTaTi complex concentrated alloy. Scr. Mater..

[22] Müller F., Gorr B., Christ H.-J., Chen H., Kauffmann A., Laube S., Heilmeier M. (2020). Formation of complex intermetallic phases in novel refractory high-entropy alloys NbMoCtTiAl and TaMoCrTiAl: Thermodynamic assessment and experimental validation. J. Alloys Compd..

[23] Lass E.A. Thermodynamics and Phase Transformations in Refractory Compositionally Complex Superalloys. Proceedings of the TMS 2023 Annual Meeting.

[24] Kube S.A., Frey C., McMullin C., Neuman B., Mullin K.M., Pollock T.M. (2024). Navigating the BCC-B2 refractory alloy space: Stability and thermal processing with Ru B2 precipitates. Acta Mater..

[25] Villars P., Okamoto H., Cenzual K. ASM Alloy Phase Diagram Database. ASM International. https://matdata.asminternational.org/apd/index.aspx.

[26] Gao Y., Guo C., Li C., Cui S., Du Z. (2009). Thermodynamic modeling of the Ru-Ti system. J. Alloys Compd..

[27] Massalski T.B., Okamoto H., Subramanian P., Kacprzak L., Scott W.W. (1990). Binary Alloy Phase Diagrams.

[28] (2023). Thermo-Calc 2023a.

[29] (2020). TCHEA4 High Entropy Alloy Database.

[30] Milenkovic S., Sabirov I., Llorca J. (2012). Effect of the cooling rate on microstructure and hardness of MAR-M247 Ni-based superalloy. Mater. Lett..

[31] Hisazawa H., Terada Y., Adziman F., Crudden D.J., Collins D.M., Armstrong D.E.J., Reed R.C. (2017). The Effect of Nb/Ti Ratio on Hardness in High-Strength Ni-Based Superalloys. Metals.

[32] Oh J.-H., Choi I.-C., Kim Y.-J., Yoo B.-G., Jang J.-I. (2011). Variations in overall- and phase-hardness of a new Ni-based superalloy during isothermal aging. Mater. Sci. Eng. A.

[33] Tatarkina A.L., Sokolovskaya E.M., Raevskaya M.V., Sokolova I.G., Esipova A.N. (1972). Investigation of the ternary system zirconium-niobium-ruthenium. Moscow Univ. Chem. Bull..

[34] Tatarkina A.L., Raevskaya M.V., Sokolova I.G., Sokolovskaya E.M. (1973). Investigation of ternary Nb-Ru-(Ti, Zr, Hf) alloys. Russ. Metall..

[35] Akhverdyan M.M., Burnasheva V.V., Rayevskaya M.V., Sokolovskaya Y.M. (1974). Investigation of phase equilibria in the system ruthenium-vanadium-hafnium. Strukt. Faz Fazovye Prevrashch. Diagrammy Sostoyaniya Met. Sist..

