# Supplementary material for: Microstructure and Phase Equilibria in BCC-B2 Nb-Ti-Ru Refractory Superalloys
Source: Materials (Basel). 2024 Nov 7;17(22):5429. doi: 10.3390/ma17225429 (PMC11595505; doi:10.3390/ma17225429)
Supplement: Supplementary file 1 [file materials-17-05429-s001.zip › materials-3218428-supplementary.pdf]

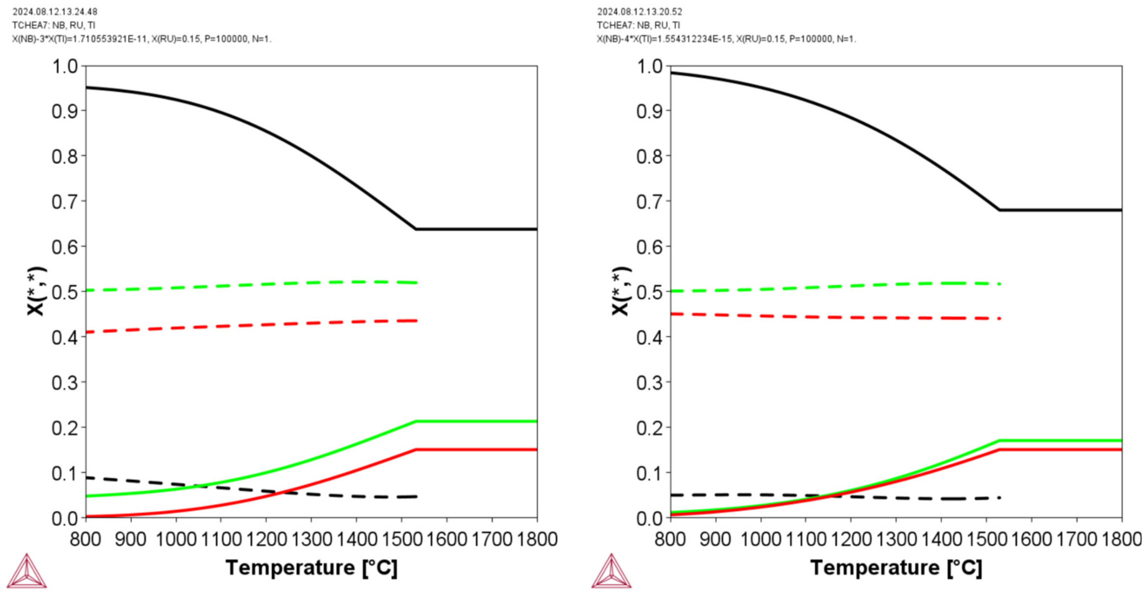

Figure S1: ThermoCalc-calculated BCC (solid) and B2 (dashed) compositions for $(Nb_3Ti)_{0.85}Ru_{0.15}$ (left) and $(Nb_4Ti)_{0.85}Ru_{0.15}$ (right) as a function of composition. Black is Nb, green is Ti, and red is Ru.

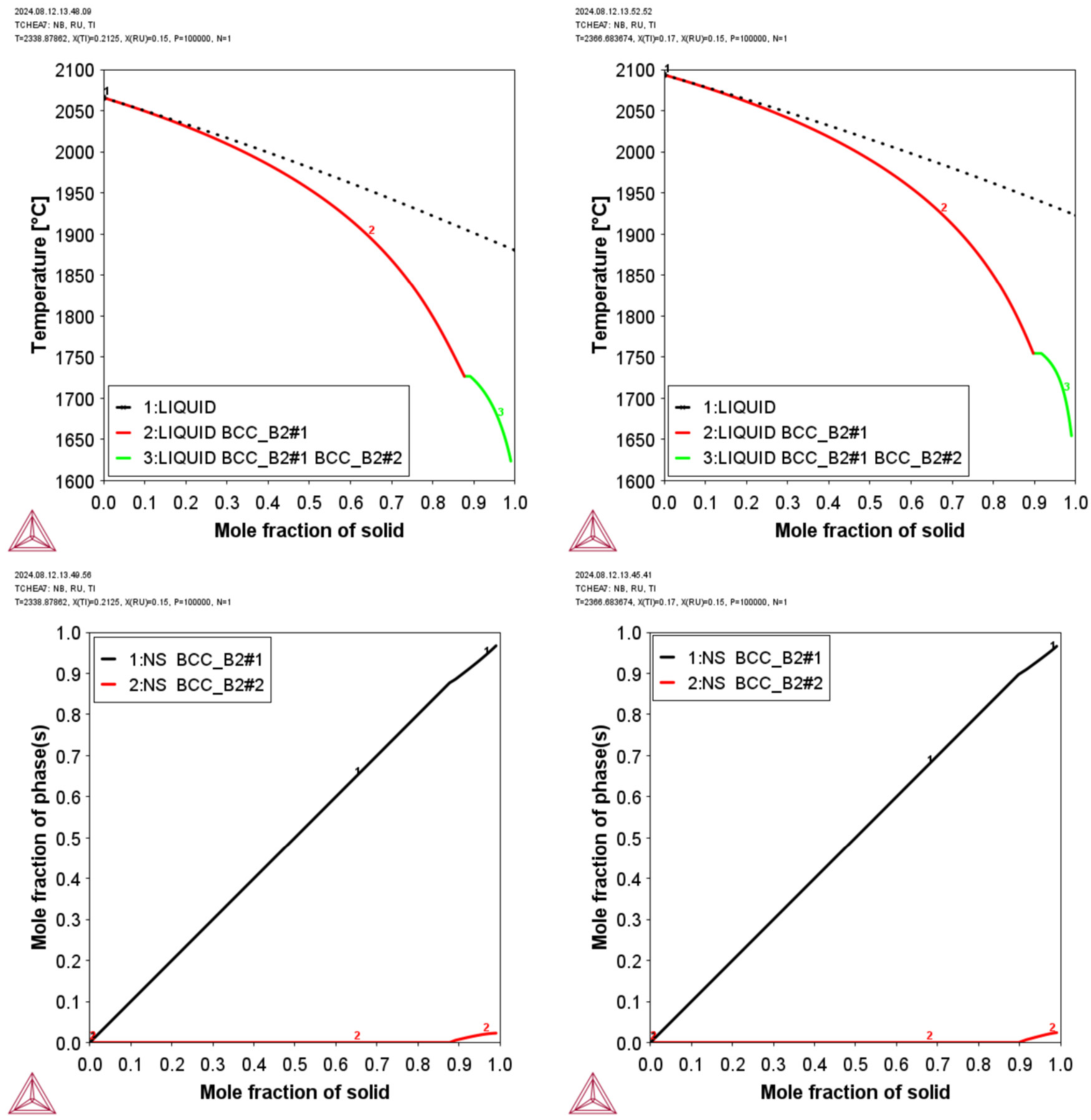

Figure S2: ThermoCalc-calculated Scheil solidification simulations for $(Nb_3Ti)_{0.85}Ru_{0.15}$ (left) and $(Nb_4Ti)_{0.85}Ru_{0.15}$ (right). Top images are temperature versus fraction solidified; bottom images are fraction of each phase versus fraction solidified. BCC_B2#2 is the RuTi-rich B2 phase.

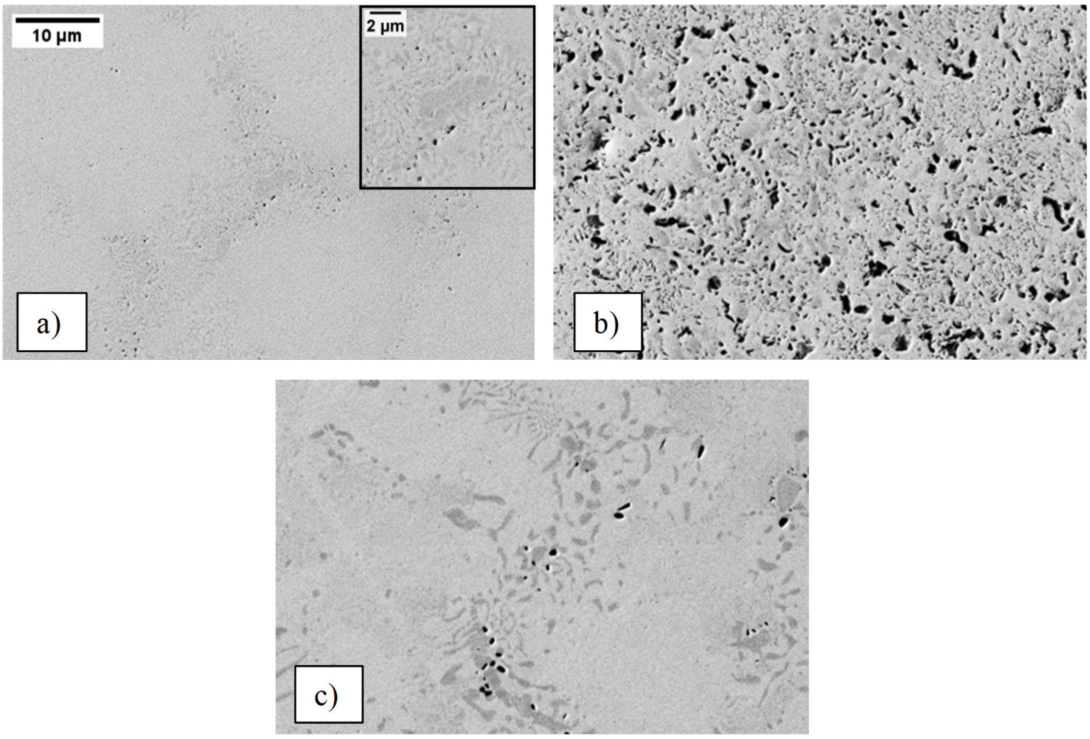

Figure S3: Micrographs of $(Nb_4Ti)_{0.85}Ru_{0.15}$ at different annealing temperatures and times: a) 900 ˚C for one week; b) 1100 ˚C for 1 week; c) 1300 ˚C for 1 day.

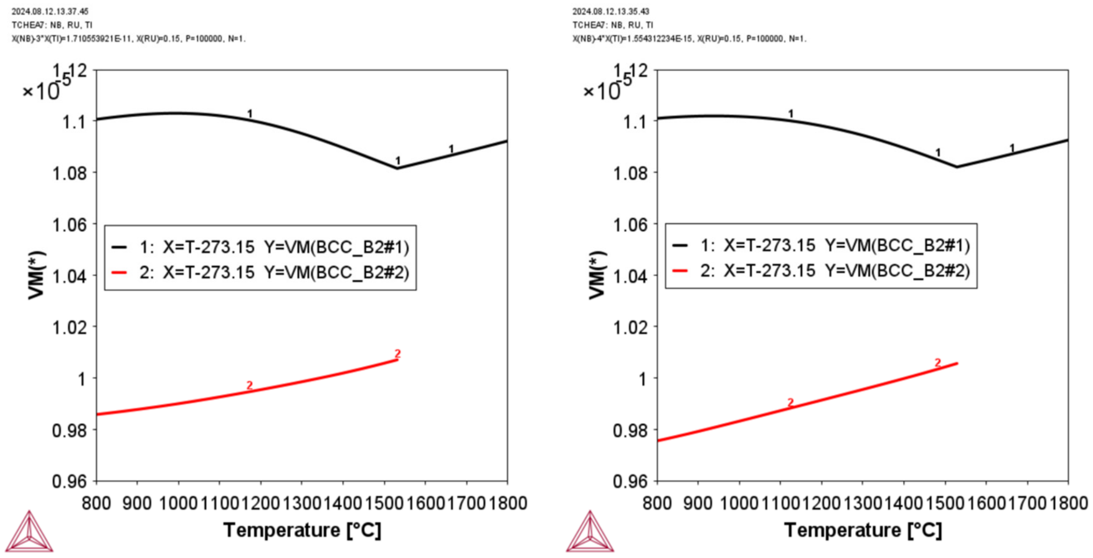

Figure S4: ThermoCalc calculated molar volumes of the BCC (BCC_B2#1) and B2 (BCC_B2#2) phases as a function of temperature for $(Nb_3Ti)_{0.85}Ru_{0.15}$ (left) and $(Nb_4Ti)_{0.85}Ru_{0.15}$ (right).
